# Quality by Design: Development of the Quality Target Product Profile (QTPP) for Semisolid Topical Products

**DOI:** 10.3390/pharmaceutics12030287

**Published:** 2020-03-23

**Authors:** Sarika Namjoshi, Maryam Dabbaghi, Michael S. Roberts, Jeffrey E. Grice, Yousuf Mohammed

**Affiliations:** 1 Therapeutics Research Group, The University of Queensland Diamantina Institute, The University of Queensland, Woolloongabba, Brisbane 4102, Australia; s.namjoshi@uq.edu.au (S.N.); m.dabbaghi@uq.edu.au (M.D.); m.roberts@uq.edu.au (M.S.R.); jeff.grice@uq.edu.au (J.E.G.); 2 School of Pharmacy and Medical Sciences, University of South Australia, Adelaide 5000, Australia; 3 Therapeutics research Centre, Basil Hetzel Institute for Translational Health Research, The Queen Elizabeth Hospital, Adelaide 5011, Australia

**Keywords:** quality by design, QbD, quality target product profile, QTPP, topical formulation, critical material attributes, CMAs, critical process parameters, CPPs

## Abstract

In recent years, the “quality by design” (QbD) approach has been used for developing pharmaceutical formulations. This is particularly important for complex dosage forms such as topical semisolid products. The first step for developing a product using this efficient approach is defining the quality target product profile (QTPP), a list of quality attributes (QAs) that are required to be present in the final product. These quality attributes are affected by the ingredients used as well as manufacturing procedure parameters. Hence, critical material attributes (CMAs) and critical process parameters (CPPs) need to be specified. Possible failure modes of a topical semisolid product can be determined based on the physiochemical properties of ingredients and manufacturing procedures. In this review, we have defined and specified QTPP, QAs, CMAs and CPPs that are required for developing a topical semisolid product based on the QbD approach.

## 1. Introduction

The skin is the largest organ of human body and the primary site of action of topical products. Semisolid dosage forms, including creams, gels, ointments, lotions, emulsions, suspensions and solutions, are the most commonly used topical formulations [1]. Quality assurance of topical semisolid products is one primary tool in guaranteeing their acceptable performance. Skin morphology and biophysiology varies greatly between individuals and between different body sites [2]. Hence, for products that need to elicit their effects within the skin, an intrinsic assertion of certain quality attributes (QAs) is imperative. Therefore, it is necessary to have quality built into the product.

A pharmaceutical dosage form, in general, will include one or more active pharmaceutical ingredients (APIs) and inactive ingredients combined together to produce a final product. The US Food and Drug Administration (FDA) defines a “high-quality drug product” as a contamination free product that can provide therapeutic benefits to the user, as specified by the label claim [3]. A formulation with obvious deficiencies may not be considered to be an effective therapeutic product and consequently may fail to be registered [4]. The most important criterion during formulation development is to meet the quality requirements. Standard quality control (QC) measures in the pharmaceutical industry include testing the final product to release and expiry specifications for the chemical, physical and microbiological parameters to account for batch to batch variations post manufacturing. These standard QC tests and measures have proved to be crucial in determining the quality of the final product post manufacturing but they are not sufficient to improve the overall quality of the product. It must be recognized that the best way to achieve high quality is to build quality into the product at every step of development, starting from the selection of the ingredients, through screening and formulation development work, scale up and establishment of manufacturing processes, including process optimization [5]. In the previous decade, the US FDA announced a new pharmaceutical regulatory concept, quality by design (QbD), which has challenged the pharmaceutical industry to design the quality of the final product instead of testing the product. The ICH guideline Q8 definition for QbD is “A systematic approach to development that begins with predefined objectives and emphasizes product and process understanding and process control, based on sound science and quality risk management” [6]. This modern aspect of product design starts with defining a list of quality requirements named the quality target product profile (QTPP). ICH Q8 defines QTPP as “A prospective summary of the quality characteristics of a drug product that ideally will be achieved to ensure the desired quality, taking into account safety and efficacy of the drug product”. These quality requirements are called quality attributes, and in order to accurately characterize the different components of QTPP, i.e., physicochemical properties, it is imperative to understand which of these can potentially be the critical quality attributes (CQAs) of a formulation. The ICH Q8 definition of CQA is “a physical, chemical, biological, or microbiological property or characteristic that should be within an appropriate limit, range, or distribution to ensure the desired product quality”. To develop a final product with desired CQAs, the quality needs to be designed into the product based on an understanding of critical material attributes (CMAs) and critical process parameters (CPPs), concepts which have been developed by the QbD approach [1]. A CMA is a physical, chemical, biological or microbiological property or characteristic of an input material that should be within an appropriate limit, range, or distribution to ensure the desired quality of output material. A CPP is defined as “A process parameter whose variability has an impact on a critical quality attribute and therefore should be monitored and controlled to ensure the process produces the desired quality”.

In this review, our focus will be to define and develop a QTPP framework for topical semisolid products by investigating all the QAs and identifying the CQAs, which are affected by CMAs and CPPs. In addition, we will investigate how correct identification and testing of these QAs using standardized methods and sensitive techniques can influence the physical and chemical stability and therapeutic performance of the product in order to mitigate performance failure in topical dermatological products.

## 2. QbD and QTPP

A new initiative entitled Pharmaceutical Current Good Manufacturing Practices for the 21st Century was announced by the FDA in 2002 to motivate the pharmaceutical industry to implement modern quality management techniques based on QbD [7]. Therefore, this model commences at the product concept stage and is used during the whole development procedure [7,8]. The fundamental principle of QbD is that quality needs to be built into the formulation by design instead of testing the formulation [4]. Performing quality control tests on manufactured products without identifying the material, process or quality attributes would have no value in reaching the high quality required [1].

QbD identifies the critical quality characteristics from the patient’s point of view and translates them into the CQAs that the final product should have. Formulations are then developed using specific CMAs and CPPs that improve manufacturing processes [4]. A comprehensive understanding of CMAs and CPPs as variables in product development is required to control them and to ensure the predefined quality of a product [4]. Design of experiment (DoE) is one such structured method that takes into account the effects of the CMAs and CPPs on the CQAs of the final dosage form [9,10]. In summary, the essential components of a successful QbD approach for topical dosage forms include

⮚ Defining a QTPP;

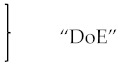

⮚ Specifying CMAs;⮚ Identifying and developing CPPs;⮚ Identifying CQAs;⮚ Controlling product and manufacturing procedures to produce final products with consistent required quality over time [11,12].

**Figure 1:**
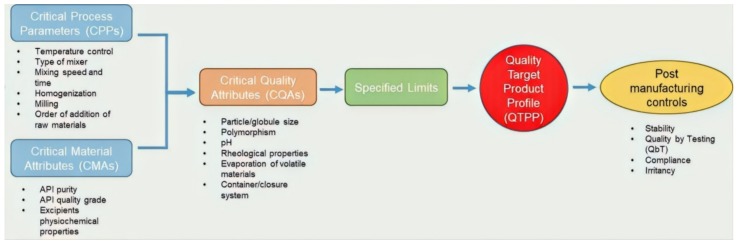
The quality by design (QbD) approach for the development of topical semisolid products. critical process parameters (CPPs) and critical material attributes (CMAs) govern the individualized QTPP for a product.

QbD offers a means of optimizing and progressing the products into and through manufacturing without meeting any additional regulatory enquiry or inspection [13]. It therefore provides self-regulatory flexibility for pharmaceutical manufacturers to accelerate the production of generic products, while at the same time maintaining quality standards [13]. QbD also leads to reductions in formulation variation, improvement of manufacturing process efficiency, and reduced timelines for market launch by decreasing additional expenditure at various stages of product development [4,14]. In the often currently used quality by testing (QbT) approach, the chemical, physical and microbiological properties of the final product are tested. In other words, quality by testing (QbT) is the way to ensure producing products with quality and manufacturing consistency, while under QbD, consistency arises from designing, developing and controlling all the manufacturing processes [11]. Figure 1 below illustrates a typical QbD approach culminating in the development of a QTPP and post manufacturing control considerations.

The QTPP for topical dosage forms is a prospective list of desired QAs that should be present in the end product [7,12]. This may include elements such as dosage form, administration route, particle or globule size, rheological behavior, drug concentration, homogeneity and uniformity, pH, in vitro drug release and permeation, microbial limits, amongst others. These can be controlled and optimized within the QbD process to produce a desired end-product [1,13,14]. Although QTPP is well defined for oral dosage forms, QTPP for topical semisolid products has not been reported yet.

To develop a QTPP for dermatological products, performance-focused CQAs should be selected, which could prove to be one of the main QbD challenges for topical semisolid products [7,14]. As such, CQAs are the characteristics of marketed products that can be altered by changing the formulation variables or manufacturing process parameters [7]. CQAs such as particle size, pH, rheological behavior, and microbial contamination play a significant role in the efficacy, safety and, specifically, the quality of the formulation [4]. Below is an example of a QTPP for a topical semisolid product (Table 1). As outlined in the draft guideline on the quality and equivalence of topical products, a patient-focused approach should be considered and, in addition to the CQAs above, the indication and disease state of skin, age appropriateness, acceptability, administration and administration site, efficacy, salt or base of the active substance, bioavailability, safety, impurities, microbial quality, physical and chemical stability and compliance should be taken into account [15].

In the subsequent sections of this review, we will outline the QAs that may influence the finished product quality taking into consideration that these attributes will vary relative to the different topical dosage forms such as solutions, gels, creams and ointments.

## 3. QAs of Topical Dosage Forms

Quality attributes (QAs) are chemical, physical, biological and microbiological characteristics that need to be defined in QTPP and presented in the final product. The QAs affecting the pharmaceutical, therapeutic and sensorial or perceptive performance of the formulation are defined as critical quality attributes (CQAs) [1]. We have outlined some key QAs below that may have a significant impact on the quality and performance of the final product.

**Particle size**: In the case of an Active Pharmaceutical Ingredient (API) suspended in a semisolid formulation, the particle size is one of the most important attributes of the product [7]. The particle size of a suspended drug in a formulation may change due to aggregation, phase separation or polymorphism over the product’s shelf life. Alterations in particle size, especially in suspensions, may lead to differences in bioavailability of the active from the semisolid product [7]. In addition, the particle size may have an effect on the perceptive attributes of the product such as smoothness and grittiness, which may determine patient compliance and acceptance.

**Globule size**: For emulsion dosage forms, globule size has been related to physical stability of the products, as well as release properties [1]. Changes in globule size may affect the amount of the drug entrapped in the globule, partitioning of active within the different phases of the product and partitioning and release into the skin. Poor control of globule size may result in phase separation, creaming or cracking which are potential failure modes of the product [16]. Globule size variations may have important implications for products packaged in multi-use containers where phase separation may lead to ultra-potent dosing for some applications and sub-potent dosing for the rest of the treatment applications. This effect may be drastic when an API is dissolved only in the oil phase of an O/W emulsion. For example, if phase separation occurs in a product packaged in a tube, the oil phase may accumulate at the tube orifice, causing majority of the drug to be consumed in the first few treatment applications [7]. On the other hand, in case of a pump dispenser, the oil phase may remain on the top and may never be applied. Preventing phase separation is therefore critical to ensure the required quality for semisolid products [7]. Manufacturing processes such as the rate of mixing of ingredients, temperature and the order of addition of excipients can have major impacts on the globule size for a given excipient combination.

**Polymorphism**: Different polymorphic forms of a drug exhibit different physiochemical properties including solubility, stability, melting point, density, texture and processing behavior [17], drug solubility and dispersion in the base may be dependent on the particular polymorphic form present. The ingredients of a formulation and their interactions with each other determine the complex formulation microstructure. Furthermore, different polymorphic forms of the same active ingredient may have different shapes and sizes that alter the microstructure of the system [18]. Changes in the polymorphic form may lead to differences in skin permeation and retention [5]. The polymorphic form of an API may be considered as a CMA. However, instability of the API in a formulation can lead to polymorphic changes, which are detrimental to the overall product performance. Further, in addition to APIs, excipients play a critical role in a topical formulation. The type, grade and source of excipients used in the manufacture of semisolid products can lead to variations in polymorphic forms [15,19].

**pH**: The solubility of some actives incorporated in topical products is pH dependent [7]. Therefore, changes in pH during a product’s shelf life may alter the solubility and bioavailability of the active, which may affect performance [7]. In addition, pH may have a significant impact on the stability of the product ingredients and a formulation’s viscosity, especially in emulsions [5]. Changing the pH can affect the zeta potential of the emulsions due to changes in the droplet size and size distribution of the emulsions [20]. If pH changes lead to decreasing the zeta potential, the emulsion stability decreases. The size and size distribution of the oil droplets, the thickness of the hydrated layer and the electrostatic interactions between the molecules can affect the viscosity of the formulation [20]. pH can affect the effectiveness of preservatives and actives. Therefore, the pH range should be limited to minimize the likelihood of detrimental effects on the actives [1]. Moreover, the pH of the formulation should be ideally adjusted to the skin physiological pH [21]. Application of a topical formulation with a pH that is markedly different from that of skin (approximately pH 5 in normal humans) may cause irritation, particularly if there is underlying skin disease [22]. Most of the topical products are adjusted to a specific pH to gain assurance that they will remain stable during their shelf life [7]. pH of the formulation is a combination of both CMAs and CPPs. The inherent nature of the API and their interactions with the excipients govern the final pH. The pH is also influenced by manufacturing processes such as the order of addition of API and excipients, whether they are added dry or in a dissolved state etc.

**Rheological properties**: Flow properties of semisolid products are key Q3 attributes. The viscosity of Newtonian fluids is independent of shear rate so as shear rate increases the viscosity remains constant, whereas for non-Newtonian materials such as topical semisolid products the viscosity is dependent on shear stress [14,23]. These non-Newtonian materials do not flow unless they have reached a critical stress level called “yield stress”. Below this point a structured material shows elastic behavior and above that point, the material’s structure will break and flow, so that its microstructure will be altered [24]. The yield stress point also correlates well with sensorial properties of topical formulations such as spreadability and ease of application [25].

Rheological characteristics have an impact on drug release from the formulation, skin penetration [26] and skin retention of the topical dosage forms [27]. By characterizing the flow behavior of a topical product, valuable insight about the microstructure of the product can be gained, which can aid in distinguishing different topical dosage forms [28]. In addition, rheological properties influence the formulation’s stability, physical appearance and performance which may change over the shelf life of the product [7]. Differences in viscoelastic properties may lead to differences in spreadability of the topical formulation leading to dissimilarity in skin feel. Patients apply topical formulations on their skin directly, and so sensorial attributes are assumed to be significant factors that can directly influence patient compliance [29,30]. Rheological properties of the formulation are a combination of CMSs and CPPs as well as the shear history of the manufactured product. A good example of this is loading of manufactured products into containers and dispensing of the products out of their packaging. A semisolid product that is dispensed form a pump experiences large shear forces that can sometimes deform their microstructure.

**Evaporation of volatile materials**: Topical formulations with different percentages of water and volatiles can be separated into different types of dosage forms [31]. For instance, as ointments are required to be retained longer on the skin, low evaporation rates are desired, which can be provided by high polyethylene glycol or mineral oil content. On the other hand, gels evaporate more rapidly due to a higher proportion of water and alcohol. Evaporation of volatiles such as water and alcohols from a formulation may lead to stiffening and changes in the microstructure of the formulation. Solvent evaporation, in addition to affecting the formulation, can also affect the API. Loss of water and volatiles can lead to changes in solubility of the active in the formulation with evaporation causing crystallization of the dissolved drug, thus changing skin retention, thermodynamic activity and penetration of the active. Therefore, the percentage of volatile excipients in topical semisolid products can be a CQA affecting performance [1]. Evaporation can also be influenced by CMAs such as type and quantity of the volatile ingredients.

**Container/closure system**: Topical semisolid products are packaged into different dispenser systems, such as jars, tubes and various types of pumps. Selection of an appropriate container/closure system is largely dependent on the dosage form and the flow properties of the product. As elaborated above, different modes of dispensing of a product may exert different shear forces on the formulation, which can affect the microstructure and therefore the performance of the product [32]. In addition, the possibility of container interaction and consequent degradation is higher in topical formulations due to their high water content. As has been pointed out [21], the FDA stability guidelines require that stability should be investigated in the actual dispenser form that is expected to go to market when pilot batches are assessed during product development.

## 4. Product Design and Development

### 4.1. CMAs

The qualitative and quantitative information of API and excipients are considered as raw material attributes [11]. The most critical part of product manufacturing is choosing a proper source of the API. Pre-formulation studies need to be performed to determine the optimal form of salt and polymorphic form of the API, evaluate its purity and quality, identify its storage temperature and shelf life, and understand its stability under different processing conditions. The grade of API impacts its physiochemical properties. For example, having various polymorphic forms is one of the resulting effects of using different grades of API, which can influence quality attributes of the final formulation. Therefore, the selection of the source of the active is fundemental for developing pharmaceutical formulations.

Since an API is mostly used at low concentrations and makes up a negligible part of the final composition, the inactive ingredients (excipients) usually define the physical characterization of a formulation [7,11]. A number of studies have shown that excipient(s) can affect the fate of an API in the skin [33,34]. As is the case for the API, the physical and chemical properties of the excipients, such as solubility, melting point, particle size, compatibility and polymorphic state are considered to be key criteria in formulation development [1]. Different grades of excipients have a substantial impact on quality attributes of the final formulation as well as the stability of the API in the product [35]. The inclusion of impurities in a particular raw material may have a detrimental effect on the stability of an API or other ingredients. Therefore, to prevent the unfavorable impact of impurities in raw materials on the performance of the final products, assessment of impurity levels is essential and sensitive analytical methods need to be developed. Another fundamental challenge during the design and development of a formulation is the compatibility of API and excipients.

### 4.2. CPPs

To design an optimal manufacturing process, all the factors including equipment, facilities, material transfer, manufacturing variables, and QTPP should be considered [11]. Mixing/homogenization time, type of mixer, temperature and mechanical energy input are the three major variables in the manufacturing of semisolid formulations. The process parameters using these linked factors need to be identified and carefully controlled to produce batches with consistent quality [1]. The impact of these process variables on product quality is discussed below.

**In process temperature control**: Choosing the correct temperature range for manufacturing is critical not only for maintaining stability of ingredients but also for dissolving and dispersing actives and excipients [3]. Temperature variation can have a considerable impact on the quality of the end product. For instance, the rate of heating or cooling of a batch may influence the consistency of the topical semisolid product. Excess heating during processing can lead to degradation of ingredients [36,37], while insufficient heat can cause product failure due to drug solubility issues [38]. The time schedule for temperature changes must be closely tailored to the process required, as excessive or rapid cooling can lead to precipitation or crystallization of solubilized ingredients or viscosity changes [36,37]. On the other hand, in some instances, rapid (shock) cooling is required immediately after heating in order to minimize grittiness from the waxes used in a formulation.

**Type of mixer**: The most commonly used manufacturing tank in the pharmaceutical industry is a stainless steel jacketed tank with an agitator. The shape, capacity and ability to maintain a desired temperature of the tank will affect the homogeneity of the product [37]. It is thus required to use a correct combination of tank, mixer blade and formulation to give a uniform distribution of the active ingredient in a batch. For example, in the case of highly viscous products, the mixer should have flexible scraper blades to remove materials from the internal walls of the tank and redistribute them into the center for mixing. The FDA recommends the use of hard plastic blades such as Teflon blades, which cause minimal damage to the tank walls. To ensure uniformity of the final product, a mixing validation procedure is undertaken on the selected tank and mixer, whereby samples are collected for analysis from the top, middle and bottom sections of the tank. The acceptance criteria for content uniformity is usually set at ±0.5% across the top, middle and bottom samples.

**Mixing speed and time**: These two factors are critical parameters that need to be accurately controlled with appropriate mixers with programmable logic controllers when manufacturing semisolid products [39]. For manufacturing gels, low shear mixing is typically required in order to maintain the viscosity of the product, while emulsification typically needs high shear rates to achieve optimum droplet size and dispersion [13,36,37]. For optimizing the mixing time, the minimum required time for dissolving the ingredients and the maximum time of mixing before which the product viscosity reduces (causing product failure) should be identified [3,13,36]. Overmixing may cause structural breakdown of polymeric gels, characterized by a drastic drop in emulsion viscosity [13]. Therefore, mixing speed and time are CPPs that can influence the QAs of the final product.

**Homogenization**: Homogenization of emulsions leads to reductions in oil globule size and aids in uniform dispersion of globules. Homogenization time is a CPP that may influence the physical stability of a formulation. [3]. In addition, excess homogenization can heat the bulk and cause instability of a thermolabile active. However, insufficient homogenization may cause insufficient mixing of the aqueous and oily phases, leading to differences in the microstructure or even phase separation of a semisolid product. Thus, using an appropriate homogenizer under vacuum pressure ensures removal of air pockets from the formulations and guarantees uniformity.

**Milling**: Milling is the reduction in the particle size of solid ingredients which can directly affect the dissolution of ingredients and have an influence on the viscosity of the final formulation [37]. The nature of the particles to be milled and the proposed size of particles guide the choice of mill. The type of mill used can affect the bulk density and particle size distribution [11,37]. The size of the mill should be large enough to de-lump the whole batch in a reasonable time, to avoid drying of elements during the milling process [37]. On the other hand, the screen size should be small enough to de-lump the ingredients properly but not too small to produce excess heating, causing materials to dry and instability of the active [37]. Another important factor is the milling speed, which can affect the particle size and subsequently affect the dissolution rate of the solid ingredients [37].

**The order of addition of raw materials**: The stage at which ingredients are introduced during production of a semisolid formulation can be important and should be well established. For example, if an API is thermolabile, it cannot be introduced during or soon after heating the surfactants in water and oil. Furthermore, to avoid precipitation, recrystallization or instability, the mixture should also be cooled to an appropriate temperature before addition of the active ingredient to the base. Preservatives such as parabens can be incorporated into the formulation just before emulsification to decrease their contact time with water-soluble surfactants at higher temperature, this prevents instability of the preservatives [36]. Thickeners (emulsion stabilizers) should be incorporated carefully when manufacturing emulsions. Amines are added to achieve optimal thickening for emulsions/gels formulated with carbomers. Depending on the order of addition, it may lead to substantial differences in the viscosity of the final product. If the amine is added in the water phase before the emulsification, the formulation viscosity will increase immediately, but as the formulation gets cooled, it thins out quickly and causes splashing out of the mixing tank [13,36,40].

## 5. Risk Assessment and Risk Control

Variations in raw material sources and proposed manufacturing processes are considered to be risk factors which can affect the critical quality attributes of the formulation and subsequently cause product failure in topical semisolid formulations [1,3]. The likelihood and potential severity of these risk factors and resulting failure modes should be identified to develop action plans towards the CMAs and CPPs, leading to mitigation of the risk factors [5]. In Table 2, we have outlined some potential risk factors, resulting failure modes and the influential CMAs and CPPs.

## 6. Conclusions

Topical semisolid products are one of the fastest growing product markets globally. Ensuring the quality and performance of these products requires well-thought-out designs in manufacturing and process. In summary, using the QbD approach for developing topical semisolid products can promote achieving the desired quality of the final product. In order to define a QTPP for a topical semisolid product, not only the QAs but also the CMAs and CPPs should be taken into account. The potential product CQAs that are derived from QTPP [31] and prior knowledge must be used as a guide for the development and manufacture of the products. Further, quality risk management can help to assess the extent of variation of the CQAs that can affect the quality and performance of the product.

## Figures and Tables

**Table 1:** Quality target product profile (QTPP) example for a topical cream.

	QTPP Elements	Target	CQAs	Justification
	Dosage form	Cream	-	-
	Route of administration	Topical semisolid product	-	Skin targeted without systemic side impacts
	Dosage strength	% w/w	-	-
	Stability	At least 12 month shelf life at room temperature	Yes	Affect the product quality
	Particle/globule size		Yes	Affect the drug permeation
	Molecular weight of Active Pharmaceutical Ingredient (API)		Yes	Affect the drug permeation
	Polymorphism		Yes	Affect the formulation uniformity and rheological properties
	pH		Yes	Affect the physiochemical stability
	Solubility		Yes	Affect the drug permeation
	Log P		Yes	Affect the drug release and skin retention
Rheological properties	Viscosity as a function of shear stress and shear rate		Yes	
	G′ (storage modulus)		Yes	
	G″ (loss modulus)		Yes	
	LVR region (linear viscoelastic region)		Yes	
	Yield stress		Yes	Affect the formulation performance
	Volatile materials content		Yes	Affect the physiochemical stability
	Container closure system		-	Affect the formulation performance
	Content uniformity		Yes	
	Microbial limitation		Yes	Affect the formulation stability and safety

G′ = Storage modulus; G″ = Loss modulus; LVR = linear viscoelastic region; Log P = partition coefficient.

**Table 2:** The possible failure modes affected by changing CMAs and CPPs.

CQAs	Related to CMAs	Related to CPPs	Failure Mode
Particle/Globule size	• Change in raw material particle sizes	• Low- or high-speed mixing • Low or high duration of mixing time	• Changes in content uniformity, drug release and dermal distribution of the drug • Patient compliance due to perceptive attributes of the product
Rheology - Viscosity -Yield stress - Tan ɣ	• Variations in viscosity of liquid/semisolid raw materials	• The order of addition of rheology modifying materials • Low- or high-speed mixing • High duration of mixing	• Changes in skin retention of the formulation and drug penetration through the skin • Changing in patient acceptability/compliance • Impact on sensorial attributes of the product
Evaporation of volatiles	• Change in proportion of volatile and non-volatile substances in the formulation	• Process temperature • High duration of mixing	• Changes in formulation microstructure (crystallization or polymorphism) • Changes in skin retention and permeation of the active • Impact on sensorial attributes of the product
Homogeneity and uniformity	• Impurity in API or excipients	• Low- or high-speed mixing • Low duration of mixing • Low temperature • Use of improper mixer type	• Differences in distribution of active through the product affecting skin permeation and therapeutic performance
Precipitation/aggregation	• Dependent on the type of emulsifier, gelling agent or volatiles	• The order of addition • High duration of mixing	• Influence on API partitioning within the formulation • Amount of drug permeating through the skin
Microbial limitations	• Contaminated materials • Ineffective preservative system	• Contaminated manufacturing and packaging equipment • Lack of or un-validated cleaning protocols for the manufacturing plant and equipment	• Microbiological contamination and both physically and chemically unstable product

CQAs = citical quality attributes, CMAs = critical material attributes, CPPs = critical process parameters, and Tan ɣ = loss tangent.

## Abbreviations

QAs	quality attributes
API	active pharmaceutical ingredient
CQAs	critical quality attributes
QbD	quality by design
QTPP	quality target product profile
ICH Q8	international conference on harmonization of technical requirements for registration of pharmaceuticals for human use. ICH harmonized tripartite guideline. Pharmaceutical development
CMAs	critical material attributes
CPPs	critical process parameters
FDA	the US food and drug administration
QC	quality control
DoE	design of experiment
QbT	quality by testing
